# Are mouse lens epithelial cells more sensitive to *γ*-irradiation than lymphocytes?

**DOI:** 10.1007/s00411-012-0451-8

**Published:** 2013-01-16

**Authors:** Kristina Bannik, Ute Rössler, Theresa Faus-Kessler, Maria Gomolka, Sabine Hornhardt, Claudia Dalke, Olena Klymenko, Michael Rosemann, Klaus-Rüdiger Trott, Michael Atkinson, Ulrike Kulka, Jochen Graw

**Affiliations:** 1 National Research Center for Environmental Health, Institute of Developmental Genetics, Helmholtz Center Munich, Ingolstädter Landstrasse 1, 85764 Neuherberg, Germany; 2 Department of Radiation Protection and Health (SG1.1), Federal Office for Radiation Protection, Oberschleißheim, Germany; 3 Institute of Radiation Biology, Helmholtz Center Munich, Neuherberg, Germany; 4 University College London Cancer Institute, London, UK; 5 Present Address: Department of Radiotherapy, University Hospital, Essen, Germany

**Keywords:** Lens epithelial cells, Lymphocytes, Gamma-irradiation, Comet assay, Gamma-H2AX assay, Radiation sensitivity

## Abstract

In this pilot study we compared for the first time the radiation sensitivity of mouse lens epithelial cells (LECs) and mouse lymphocytes. We freshly prepared LECs and lymphocytes and irradiated them with *γ*-rays (^137^Cs; doses ranging from 0.25 to 2 Gy). DNA damage and repair were evaluated by alkaline comet assay and *γ*H2AX foci assay. Using the comet assay, we observed a dose-dependent increase in DNA damage in both cell types. The faster formation of single- and double-strand breaks in LECs of C57BL/6 mice at doses below 1 Gy needs to be confirmed in other mouse strains. Immunofluorescence for *γ*H2AX foci showed a higher degree of lesions in LECs from C57BL/6J mice compared to those of JF1 mice and to lymphocytes of both strains. Correspondingly, repair of DNA damage proceeded faster in LECs of C57BL/6J mice compared to LECs of JF1 mice and lymphocytes of both strains. It is obvious that the lymphocytes of both strains repaired DNA lesions more slowly than the corresponding LECs. In conclusion, our results demonstrate that LECs of C57Bl/6 mice show a steeper dose–response than lymphocytes in both types of experiments. It shows that both test systems are able to be used also at doses below 0.25 Gy. The observed difference in DNA repair between the LECs from C57BL/6J mice compared to the LECs from JF1 mice and to the lymphocytes of both strains warrants further experiments to identify the underlying molecular mechanisms.

## Introduction

For a long time, it has been known that ionizing radiation causes opacification of the ocular lens (cataracts), and the lens is usually considered as a radiation-sensitive tissue (for a review see Ainsbury et al. 2009). This concept of a particular radiation sensitivity of the ocular lens is based primarily upon its unique cellular architecture: the life-long dividing epithelial cells at the anterior (and most exposed to the exterior) lens epithelial cells (LECs), and the terminally differentiated lens fiber cells, which degrade their cellular organelles (e.g., cell nucleus and mitochondria), but remain persisting in the lens in an onion-like structure for the entire life (for a recent review of lens cell differentiation see Bassnett, 2009).

There are only a few reports on the underlying molecular mechanisms in radiation-induced cataract formation. For very high doses (11 Gy), the participation of DNA damage followed by the formation of reactive oxygen species, DNA adducts and chromosomal rearrangements have been described as causative (Wolf et al. 2008; Pendergrass et al. 2010). However, these doses remain relevant to the lens only in particular therapy schedules of head-and-neck cancers. Today, the consequences for cataract formation of doses below 1 Gy are much more challenging, since recent epidemiological studies on interventional radiologists (Jacob et al. 2012) as well as data from mouse experiments indicate a broad variation in the frequency of cataract formation after low-dose ionizing radiation, which might be based upon differences in the genetic background (Worgul et al. 2005). It indicates that the personal risk for cataract formation after low-dose ionizing radiation depends considerably on the individual genetic predisposition. In contrast, at higher doses the variation of the genetic background is less important compared to the general and severe damaging effects of ionizing radiation.

Therefore, we investigated in a first set of experiments whether the radiation sensitivity and repair capacity of DNA damages of LECs are comparable to that of lymphocytes. In cell cultures, such questions can be answered much faster than in lifetime studies using model organisms like mice. However, for clinical aspects like cataract formation, such animal experiments are finally indispensable. We tested radiation sensitivity by the comet assay and DNA repair capacity by the *γ*H2AX foci formation. Lymphocytes have been well investigated for their radiation sensitivity in many organisms including mice and humans (UNSCEAR Report, 2006). In a second step, we compared also the *γ*H2AX foci formation in both cell types of two different mouse strains: C57BL/6J mice were used as one of the widely used reference strains (http://www.informatics.jax.org) and compared with cells of Japanese Fancy Mice (strain designation: JF1). JF1 mice have been demonstrated recently to produce F1 offspring that were more resistant to radiation-induced thyroid lesions as compared to C57BL/6J and BALB/c hybrid mice (Dalke et al. 2012).

Here we demonstrate for the first time a comparison of radiation sensitivity of LECs derived from two different mouse strains particularly with regard to lymphocytes. To relate our results to previous data on human lymphocytes (Rössler et al. 2006), we used doses of 0.5, 1 and 2 Gy. Additionally, one lower dose (0.25 Gy) was used to find out whether it is possible to apply the chosen assay systems also to doses below 0.5 Gy. Within this dose range (0.25–2 Gy), our results indicate cell-type-specific differences between the mouse strains tested here. Our results open also novel possibilities to unravel cell-type-specific differences and the underlying molecular mechanisms for better understanding the variation in the frequencies of radiation-induced cataracts among humans.

## Methods

### Mice and cell culture

Mice (C57BL/6J and JF1) were kept under specific pathogen-free conditions at the Helmholtz Center Munich. The use of animals was in accordance with the German Law of Animal Protection, the ARVO Statement for the Use of Animals in Ophthalmic and Vision Research, and the tenets of the Declaration of Helsinki.

For preparation of LECs, adult male mice (age: 7–10 weeks) were killed by CO_2_, the eyeballs were removed, and the lenses were prepared. The lens capsule with the attached lens epithelium was removed from the lens in suspension medium (medium 199 containing 0.1 % BSA, 100 U penicillin/ml, 100 μg streptomycin/ml and 2.5 μg amphotericin B/ml). The capsules were collected in a 1.5-ml test tube and centrifuged for 5 min at 2,000 rpm. The suspension medium was removed carefully, and 300 μl trypsin (0.05 %) was added. After shaking for 10 min, the cells were washed once in PBS; after centrifugation, the cells were re-suspended in suspension medium (including FGF2; 100 ng/ml), transferred into 24-well plates and incubated at 37 °C with 5 % CO_2_. To attach cells to the surface of the plates, they were washed with 100 % FBS (fetal bovine serum) before putting cells into them. For *γ*H2AX foci assay, the LECs grew in the wells on coverslips (IBIDI, Martinsried, Germany).

For the preparation of resting lymphocytes, 4–5 ml heparin blood was collected from two to three adult male mice, and peripheral blood mononuclear cells (PBMC) were separated via a Biocoll gradient using Greiner Bio-One’s Leucosep tubes following the instruction manual. For the comet assay, cells were trypsinized from the 24-well plate, washed and diluted in 0.9 % NaCl to a concentration of 50,000 cells per slide. For the *γ*H2AX assay 200,000 lymphocytes were adjusted in 100 μl RPMI medium (Biochrom, Berlin, Germany) prior to radiation. For both assays, the lymphocytes are not stimulated for cell divisions.

Standard chemicals were from Serva (Heidelberg, Germany), Sigma-Aldrich (Taufkirchen, Germany), Merck (Darmstadt, Germany) or Carl Roth (Karlsruhe, Germany), if not otherwise mentioned.

### Radiation

Irradiation of LECs and lymphocytes for doses of 0.25, 0.5, 1 and 2 Gy was performed using the ^137^Cs-source HWM-2000 (Markdorf, Germany) at a dose rate of 0.5 Gy/min. For the comet assay, the radiation was performed in suspension (10 μl suspension of LECs or 100 μl suspension of lymphocytes), and cells were kept on ice. For the *γ*H2AX assay, cells were irradiated as adherent monolayers at room temperature. DNA repair kinetics were measured by incubation of the irradiated samples at 37 °C for defined time intervals (comet assays: 0, 5, 15, 60 min; *γ*H2AX assay: 1, 4, 24 h) to allow DNA repair before conducting the assay procedure.

### Comet assay

After irradiation, the cells were mixed with an equal volume of agarose and spread in a thin layer on glass slides to perform alkaline comet assay as described previously (Gomolka et al. 2005; Rössler et al. 2006). Slides were scanned using a fluorescence microscope (Axioplan 2; Carl Zeiss, Jena, Germany). For automatic analysis, we used the Metafar-4 software (MetaCyte v.3.1.3; MetaSystems, Altlussheim, Germany) counting 200 cells per slide. For LECs, the comet data are based upon three independent primary LEC cultures each representing a pool of 20 lenses (20 lenses per one single dose with 4 time points and 2 slides; i.e., 100 lenses for the entire experiment); for lymphocytes, three sets of independent experiments (i.e., different lymphocyte preparations from different mice) have been performed for C57BL/6J mice, and two for JF1 mice.

### *γ*H2AX foci assay

LECs were incubated at 37 °C for 30 min to allow phosphorylation of histones. Subsequently, cells were fixed in 4 % paraformaldehyde (PFA) for 10 min, washed in PBS three times and finally blocked (PBS containing 1 % normal Donkey serum and 0.1 % Triton X-100) for 1 h at room temperature. The coverslips were rinsed in wells three times with PBS, subsequently with 160 μl blocking solution, and incubated overnight with pH2AX antibody (Active Motive, La Hulpe, Belgium) diluted 1:500 in blocking solution at 4 °C to allow for binding to the phosphorylated Ser139 of histone H2AX. All buffer changes were done very gently using a pipette with a wide opening to preserve the cells. The coverslips were washed in PBS three times and incubated with an anti-rabbit-IgG secondary antibody (1:500; Cy3 conjugated; Jackson Immuno/Dianova, Hamburg, Germany) for 1 h at room temperature. Afterward, the cells were washed in PBS three times and incubated with DAPI (Sigma-Aldrich, Taufkirchen, Germany) for 10 min. Finally, cells were washed once more in PBS and dried on air in the dark. For mounting we used a drop of mounting solution aqua Polymount (Polysciences, Eppelheim, Germany). The fluorescence analyses were performed using a fluorescence microscope (Leica, Wetzlar, Germany) with filters for DAPI and Cy3.

In LECs, the *γ*H2AX foci were counted by eye in blinded fashion in randomly chosen cells. Only nuclei, which were in accordance with the standard criteria, were considered for evaluation, but cells with apoptotic features were rejected. The pictures were taken using the Leica Application Suite Advanced fluorescence software, and at least 100 nuclei were counted or each sample.

Lymphocytes were treated and analyzed in a slightly different way. Prior to fixation, cells were spun on top of slides for 5 min/500 rpm using a Cytospin centrifuge (Hettich, Tuttling, Germany). Cells were fixed in 2 % PFA for 15 min, washed 3 times for 5 min each in PBS/0.15 % Triton and blocked with PBS/1 % BSA 3 times for 10 min each. Cells were covered with 75 μl of the primary antibody (*γ*H2AX; New England Biolabs, Frankfurt a. M., Germany) diluted 1:200 in PBS/BSA and incubated in a humid chamber at 4 °C overnight. After washing with PBS (5 min), PBS/Triton (10 min), PBS (5 min) and PBS/BSA (7 min), cells were incubated with 75 μl of the secondary antibody (Alexa Fluor 555; New England Biolabs) diluted 1:1,000 in PBS/BSA for 45 min at 4 °C. Again, cells were washed in PBS/Triton 2 times for 5 min and in PBS 2 times for 10 min. For counterstaining, cells were incubated with Hoechst-33342 (Sigma-Aldrich, Taufkirchen, Germany) for 2 min and washed in PBS two times. Prior to microscopic analysis, cells were covered with 16 μl Vectashield (Vector Laboratories, Burlingame, USA) and sealed with a coverslip. For foci analysis, an automated scanning and analysis system was used (Axioplan 2; Carl Zeiss, Jena, Germany; Metafer4, MetaSystems-Altlusheim, Germany).

For LECs, the foci data are based upon two independent primary LEC cultures (each representing a pool of 10 lenses) for C57BL/6J and one for JF1 mice; for lymphocytes, two sets of independent experiments (i.e., different lymphocyte preparations from different mice) have been performed for C57BL/6J and JF1 mice.

### Statistics

The percentage-of-DNA values from the comet assay and the spot counts from the *γ*H2AX assay were averaged over the cells of each slide. Group-specific means were calculated for the combinations of cell type and strain. These calculations were performed with mixed-effects models (Pinheiro and Bates 2000), taking into account the grouping of slides within biological replicates (mice for the lymphocyte data and cell pools for the lens data). Mixed-effects models were also applied for ANOVA and ANCOVA calculations. The effect of incubation time was studied in ANOVAs treating the incubation time as an ordered factor. The dose effect for the initial time point was studied in two versions: assuming a linear effect and treating dose as an ordered factor.

In two- and three-way ANOVAs and ANCOVAs, first the highest possible interaction was tested for significance. In case of a significant interaction effect, analyses were performed within subgroups. Statistical testing was performed with 0.05 level. For all calculations, the R software with the nlme package was used (Pinheiro et al. 2012; R Development Team, 2011).

## Results

### Characterization of mouse lens epithelial cells in culture

**Fig. 1:**
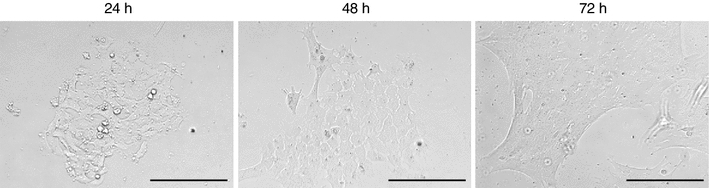
Mouse lens epithelial cells in culture. Lens epithelial cells (LECs) of 10 weeks old C57BL6/J mice in culture after 24, 48 or 72 h (*bar* = 200 μm)

Primary cultures of C57BL/6J mouse LECs are quite heterogeneous in size after 24 h; they become more homogenous within the first 72 h and began at that time to elongate (Fig. 1). The number of living cells (as indicated by the non-uptake of trypan blue) remains roughly constant during the first 4 days, indicating that most of the LECs are not dividing. This is in line with the previous reports showing that these cells in vivo are arrested in G_1_ phase (Muggleton-Harris and Higbee, 1987). Moreover, apoptotic or necrotic cells were still present, which does not allow a statement on the amount of apoptotic or necrotic cells due to radiation. However, when LECs are kept in culture for longer time, they grow regularly as a monolayer as described previously (Muggleton-Harris and Higbee, 1987). To be very close to the in vivo epithelial state of the lens cells, we used LECs after 3 days in culture in all assays described here.

### DNA damage in lens epithelial cells and in lymphocytes analyzed by the comet assay

**Fig. 2:**
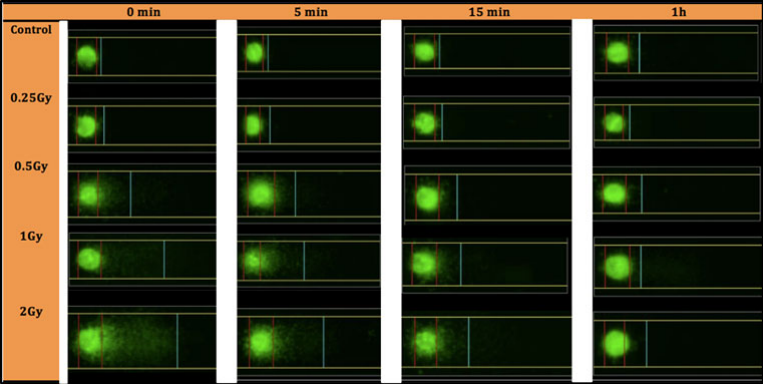
Examples of lens epithelial cells after irradiation and comet assay. Examples of DNA damage after comet technique in mouse lens epithelial cell nuclei after irradiation with ^137^Cs (0.25–2.00 Gy) and repair time of 0–60 min. *Bars* describe the amount of DNA in head (*red*) and DNA in tail (*blue*)

**Fig. 3:**
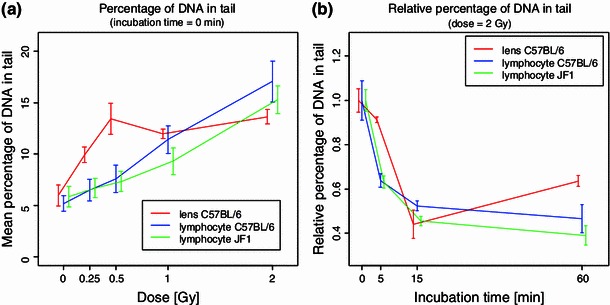
Comet assay: DNA damage in lens epithelial cells and lymphocytes after irradiation. **a** The damage of DNA (as indicated by the tail length in the comet assay) is dose dependent. Data are presented as mean ± SEM (*n* between 2 and 5). **b** After radiation with 2 Gy, cells are allowed to repair DNA up to 60 min. The initial DNA damage is set to 1 (i.e., relative percentage of DNA in tails are shown); the *graph* indicates clearly that the two cell types do not show major differences in their repair capacity. Data are presented as mean ± SEM (*n* between 2 and 5)

The DNA damage caused by *γ*-ray (between 0 and 2 Gy) was measured in mouse LECs and lymphocytes using a modification of the “comet assay” originally described by Singh et al. (1988). Representative examples of the DNA in the nuclei of LECs and the correspondingly damaged DNA are given in Fig. 2. It is obvious that in general the amount of DNA damage (indicated by parameter “% DNA” in the comet’s tail) increases with dose (Fig. 3a). As exception, the amount of initial DNA breaks in LECs did not further increase at doses above 0.5 Gy. Further experiments are necessary to understand the underlying mechanisms. Because of the limitations in the breeding capacity of the JF1 mice and because of the particular high number of LECs necessary for the comet assay, we could not compare LECs from the two strains.

In the dose range 0–0.5 Gy, the dose–response is linear, and the DNA of LECs is obviously more degraded than the DNA of lymphocytes. To have a closer look at the response of the different cell types in this dose range, we performed an analysis of covariance (ANCOVA) and tested for interaction effect (i.e., for group differences between slopes of the dose–response lines). The slopes of the increase in DNA damage are significantly different between the three groups (*p* = 0.0004 for the interaction effect in the ANCOVA). However, in subcomparisons we saw that between the lymphocytes of the two different strains, the slopes are not significantly different (*p* = 0.33), that is, the significance of slope differences is due to the LECs which are the most sensitive cells.

If the cells were incubated up to 1 h and thereby allowed to repair their DNA breaks, the percentage of DNA in the tail decreased with increasing incubation time (Fig. 3b). Since DNA repair was always related to the amount of the initial damage (the maximal percentage of “DNA in tail” was set to 1; Fig. 3b), differences between methods of quantification could be minimized by calculating the relative remaining damage (dividing all values by the corresponding initial value). In general, the differences between lymphocytes and LECs in terms of DNA repair kinetics observed in the comet assay have to be confirmed in additional experiments.

### DNA repair in lens epithelial cells (*γ*H2AX assay)

**Fig. 4:**
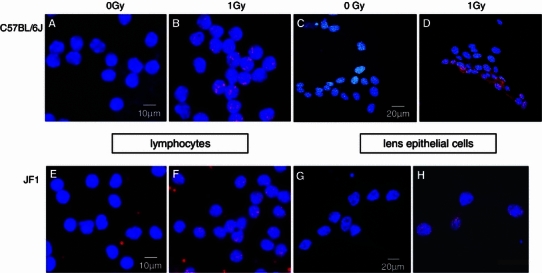
*γ*H2AX staining in mouse lens epithelial cells and lymphocytes after irradiation. Examples of immunofluorescence are given for the staining of *γ*H2AX in mouse cells without (**a**, **c**, **e**, **g**) and after 1 Gy irradiation and repair time of 1 h (**b**, **d**, **f**, **h**). *Blue* staining of cell nuclei with DAPI. *Red* staining with antibody against *γ*H2AX (coupled with Cy3). Magnification ×40; *bars* indicate size. *Upper row* (**a**–**d**): cells from C57BL/6J mice, *lower row* (**e**, **f**): cells from JF1 mice; *left* lymphocytes (**a**, **b**, **e**, **f**); right, lens epithelial cells (**c**, **d**, **g**, **h**)

**Fig. 5:**
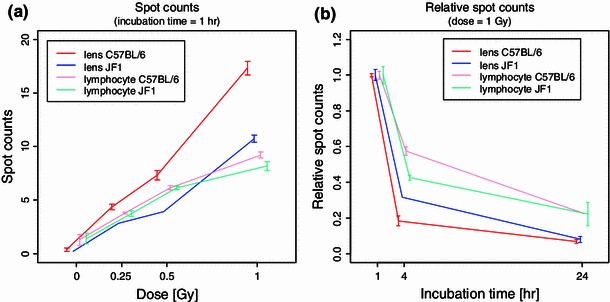
DNA repair process after radiation at 1 Gy. **a** The number of *γ*H2AX foci increases in a dose-dependent manner. The LECs from C57BL/6J mice show more foci than the other cells. Data are presented as mean ± SEM (*n* between 2 and 5; except for the JF1 LEC data at the doses of 0.25 Gy and 0.5 Gy with *n* = 1). **b** The number of *γ*H2AX foci after irradiation at 1 Gy and incubation time of 1 h was set to 1 (i.e., relative spot counts are shown); it decreases with increasing incubation time, which was allowed for 24 h. Repair proceeds faster in the LECs compared to lymphocytes; the strain effect on repair is different between the cell types (*p* value for interaction effect in the three-way ANOVA; *p* = 0.0096). Data are presented as mean ± SEM (n between 2 and 5; except for the JF1 LEC data at the incubation time of 4 h and the C57BL/6 lymphocyte data at the incubation time of 24 h with *n* = 1)

The phosphorylation of histone H2AX was the first histone modification demonstrated to be induced by DNA damage [mainly DNA double-strand breaks Löbrich et al. (2010)]. This epigenetic marking of the DNA at the breakage sites is the first step initiating DNA repair. When DNA repair is finished, H2AX has to be dephosphorylated to allow restoration of the epigenome to the predamage status (for a recent review see Lukas et al. 2011). This can be shown by the decline of foci number with time. We tested this by incubation of the cells for different, defined repair times. Representative examples of residual *γ*H2AX staining in irradiated LECs and lymphocytes of both mouse strains (C57BL/6J and JF1) are given in Fig. 4. After ^137^Cs irradiation, the number of foci in the nuclei of the irradiated cells increases in a dose-dependent manner, in both LECs and lymphocytes from both mouse strains (Fig. 5a). Similar to the comet assay, the LECs from C57BL/6J mice show more DNA damage indicated by the number of foci at each radiation dose tested than the lymphocytes of both strains and the LECs from JF1 mice. In all cases, the dose effect is significant for the lowest dose (0.25 Gy; *p* values below 10^−4^).

A more detailed comparison of the DNA repair is given in Fig. 5b on the relative damage. The LECs of C57BL/6J mice repair their DNA faster than all other cells; the LECs from JF1 mice are closest to them. The lymphocytes of both strains repair more slowly than the corresponding LECs; however, the differences between the strains are in opposite directions: JF1 repair is slower in LECs but faster in lymphocytes, indicating a different regulation of at least two processes in various tissues and strains.

It should be noted that for the *γ*H2AX assay different antibodies and microscopes for the evaluation had been used. Nevertheless, the differences between LECs and lymphocytes are considered to be reliable, since inter-laboratory comparisons of the *γ*H2AX assay under different laboratory conditions lead to very similar results (Ute Roessler, personal communication). Moreover, since DNA repair was always based relative to the amount of the initial damage (the maximal initial spot count was set to 1; Fig. 5b), differences between methods could be minimized.

## Discussion

The lens is recognized as a tissue, which is sensitive to ionizing radiation (Ainsbury et al. 2009); the clinical consequence of ionizing radiation is opacification of the lens (cataract) resulting in impaired vision making it finally necessary to extract the opaque lenses. Age is one of the most important “risks” for cataracts; at the age of ~70 years, roughly 25 % of a Caucasian population suffers from cataract; besides age, female sex, diabetes and hypertension are important risk factors (Graw et al. 2011 and references therein). In addition, genetic factors influencing the functional integrity of the human lens are important to keep it transparent even at higher age; such factors include SNPs in *GJA8* (Graw et al. 2011) or *EPHA2* (Shiels et al. 2008). The interaction between risk factors (such as those which are genetically determined) and radiation exposure has not been sufficiently studied, so far.

In mice, Gajewski et al (1977) demonstrated that the time for cataract formation is strongly dependent on the age, when mice received irradiation (300 R of X-ray): Mice irradiated 1–3 days after birth develop cataracts (stage 1+) after ~50 days (median). In contrast, if mice were irradiated at the age of 3 weeks (or older, up to 52 weeks of age), cataracts (stage 1+) developed after ~200 days (median). It indicates that the age of mice when we prepared LECs (between 7 and 10 weeks of age) would not affect radiation sensitivity.

The genetic susceptibility for radiation-induced cataracts (in mice) appears to be much more important at low-dose exposure of the lens than at high doses (Worgul et al. 2005). These authors investigated the difference in cataract formation in mice not only at different doses, but also in genetically diverse mice: They used hetero- and homozygous *Atm* mutants being characterized by deficits in DNA repair. After 8 Gy to lenses of *Atm* mutant mice, cataracts appeared rapidly and at the same rate in wild-type and mutant mice. However, after 0.5 Gy, cataracts appeared later in life, but the heterozygous mutants developed cataracts earlier than irradiated wild-type mice. Therefore, our experiments were designed to address the question of the genetic component(s) contributing to the risk of radiation-induced cataracts.

Since cataract formation after low-dose irradiation takes several months, we have chosen primary LECs to analyze their radiation sensitivity and DNA repair capacity. To test whether we can identify in this test system any differences among their inherent radiosensitivity, we used two genetically far distant mouse strains to address this question. Moreover, to make the lens studies comparable to previous radiobiological studies, we used additionally lymphocytes derived from the same strains, that is, C57BL/6J and JF1. Thus, our results demonstrate for the first time a difference in radiation sensitivity between LECs and lymphocytes of two genetically far distant mouse strains. As a first approximation, the radiation sensitivity at the level of DNA damage of LECs is in the same order of magnitude as of lymphocytes (due to the large amount of LECs necessary for the comet assay, the comparison between LECs of the two strains could be performed in C57Bl/6 mice only). At doses <0.5 Gy, LECs from C57BL/6J are approximately three times as sensitive as lymphocytes. Similarly, the LECs of C57BL/6J (and of JF1 mice) origin showed a faster DNA repair compared to other cells. Therefore, from both types of experiments, we can conclude that LECs are more sensitive to radiation than lymphocytes. However, the molecular mechanisms underlying these discrepancies remain unknown: Since the genetic differences between the two mouse strains (C57BL/6J and JF1) are not yet elaborated, it is quite difficult to speculate about these mechanisms. In particular, it would be necessary to sequence the JF1 genome to identify differences in genes encoding proteins involved in DNA repair; finally, their enzymatic activities have to be compared biochemically.

We are also aware that—because of some cell- and tissue-specific needs—different methods have been used to propagate and assay the LECs and the lymphocytes. This might influence the absolute data given in Figs. 3a and 5a. To overcome this problem, we added for both experiments the relative data given in Figs. 3b and 5b: For each assay, cell type and dose point, we set the maximum number of damage as 1 and calculated the relative repair independent of the absolute number.

The higher sensitivity of LECs from C57BL/6J mice (but not from JF1 mice), relative to lymphocytes from both strains, shows that genetic differences in radiation response can be very tissue specific, and lymphocytes are not necessarily a general indicator of congenital factors. The data presented here suggest the possibility to address the question of genetic susceptibility to radiation-induced cataracts on a cellular level. Even if these cellular assays cannot replace the experiment with cohorts of living mice, it might indicate which strains of mice should be used for such experiments to cover the full range of radiation sensitivity in the lens. Such future experiments will also include cells from female mice (and female cohorts of living mice), because previous epidemiological studies in humans showed clearly that females develop earlier and more frequently cataracts (Graw et al. 2011 and references therein). However, for this pilot study, we used only male mice to restrict the number of different parameters to those we are mainly interested in.

The dose range used in this pilot study demonstrated that the experiments can be extended to lower doses than 0.25 Gy as it was used here. In future experiments, we will expand our investigations to doses lower than 0.25 Gy, but also to other wild-type mouse strains as well as to mouse mutants with known genetic defects. Such experiments will allow us to analyze in more detail differences in radiation sensitivity and the underlying molecular DNA repair mechanisms.
